# Amentadione from the Alga *Cystoseira usneoides* as a Novel Osteoarthritis Protective Agent in an Ex Vivo Co-Culture OA Model

**DOI:** 10.3390/md18120624

**Published:** 2020-12-07

**Authors:** Nuna Araújo, Carla S. B. Viegas, Eva Zubía, Joana Magalhães, Acácio Ramos, Maria M. Carvalho, Henrique Cruz, João Paulo Sousa, Francisco J. Blanco, Cees Vermeer, Dina C. Simes

**Affiliations:** 1 Centre of Marine Sciences (CCMAR), University of Algarve, 8005-139 Faro, Portugal; naraujo@ualg.pt (N.A.); caviegas@ualg.pt (C.S.B.V.); 2 GenoGla Diagnostics, Centre of Marine Sciences (CCMAR), University of Algarve, 8005-139 Faro, Portugal; 3 Department of Organic Chemistry, Faculty of Marine and Environmental Sciences, University of Cadiz, 11510 Puerto Real (Cádiz), Spain; eva.zubia@uca.es; 4 Unidad de Medicina Regenerativa, Grupo de Investigación en Reumatología (GIR), Instituto de Investigación Biomédica de A Coruña (INIBIC), Complejo Hospitalario Universitario de A Coruña (CHUAC), Sergas, 15006 A Coruña, Spain; joana.cristina.silva.magalhaes@sergas.es (J.M.); fblagar@sergas.es (F.J.B.); 5 Agrupación Estratégica CICA-INIBIC, Universidade da Coruña (UDC), 15006 A Coruña, Spain; 6 Centro de Investigación Biomédica en Red (CIBER), 28029 Madrid, Spain; 7 Department of Orthopedics and Traumatology, Hospital Particular do Algarve (HPA), 8005-226 Gambelas-Faro, Portugal; acacioramos@gmail.com (A.R.); mariamiguelfc@gmail.com (M.M.C.); cruzhenrique@hotmail.com (H.C.); jprsous@gmail.com (J.P.S.); 8 Cardiovascular Research Institute CARIM, Maastricht University, 6229 EV Maastricht, The Netherlands; cees.vermeer@outlook.com

**Keywords:** osteoarthritis, amentadione, preclinical osteoarthritis models, marine compounds, *Cystoseira usneoides*, inflammation, mineralization, chondrocytes, synoviocytes, cartilage explants

## Abstract

Osteoarthritis (OA) remains a prevalent chronic disease without effective prevention and treatment. Amentadione (YP), a meroditerpenoid purified from the alga *Cystoseira usneoides*, has demonstrated anti-inflammatory activity. Here, we investigated the YP anti-osteoarthritic potential, by using a novel OA preclinical drug development pipeline designed to evaluate the anti-inflammatory and anti-mineralizing activities of potential OA-protective compounds. The workflow was based on in vitro primary cell cultures followed by human cartilage explants assays and a new OA co-culture model, combining cartilage explants with synoviocytes under interleukin-1β (IL-1β) or hydroxyapatite (HAP) stimulation. A combination of gene expression analysis and measurement of inflammatory mediators showed that the proposed model mimicked early disease stages, while YP counteracted inflammatory responses by downregulation of COX-2 and IL-6, improved cartilage homeostasis by downregulation of MMP3 and the chondrocytes hypertrophic differentiation factors Col10 and Runx2. Importantly, YP downregulated NF-κB gene expression and decreased phosphorylated IkBα/total IkBα ratio in chondrocytes. These results indicate the co-culture as a relevant pre-clinical OA model, and strongly suggest YP as a cartilage protective factor by inhibiting inflammatory, mineralizing, catabolic and differentiation processes during OA development, through inhibition of NF-κB signaling pathways, with high therapeutic potential.

## 1. Introduction

Osteoarthritis (OA) is a prevalent degenerative arthritic disease, a chronic condition that causes pain and disability among elderly patients [1,2]. An estimated 10% to 15% of all adults aged over 60 have some degree of OA [3], with prevalence doubling since the mid-20th century [4]. OA has been defined as a “whole joint” and multifactorial disease, characterized by synovial inflammation, progressive loss of articular cartilage and remodeling of the underlying bone [5,6].

Although OA physiopathology is still not completely understood, chronic inflammation is known to play a critical role in disease development and progression, with accumulating evidence supporting the association between OA pathology and different markers of inflammation [7]. OA cartilage and synovium overexpress cytokines and pro-inflammatory mediators that stimulate the accumulation of proteolytic enzymes, aggrecanases and matrix metalloproteinases (MMPs) responsible for the extracellular matrix (ECM) degradation, and for mediating detrimental effects through innate immunity signals [8,9]. In particular, MMP3 is known to mediate the integrity of various constituents of the ECM, such as collagens (types II, III, IV, V, VII, IX, X), fibronectin, elastin, proteoglycans, directly or through the activation of other pro-MMPs and pro-TNFα, in OA [10]. This molecular condition together with chondrocyte differentiation into a hypertrophic phenotype, result in loss of the ability to restore the ECM with consequent cartilage degradation. Basic calcium phosphate (BCP) deposition in the cartilage and synovial membrane is closely associated with OA inflammation, and contributes to local tissue damage and failed tissue repair, further intensifying hyaline articular cartilage loss and progressive joint deterioration [11,12,13].

Current osteoarthritis prevention and treatment are still very limited and unsatisfactory, with therapeutics focused mainly in drugs which improve pain or symptoms, such as topic and oral nonsteroidal anti-inflammatory drugs, acetaminophen, and opioids [14]. Although there are some advances in the design of new molecules to target cartilage repair and bone, or to treat inflammation and pain, at present, no effective OA drugs have yet been approved [15], making the search for new potential molecules a priority to overcome the growing burden of OA.

In addition, the lack of reliable models able to simulate the physiologic OA scenario, contributes to slow down the discovery of novel preventive or therapeutic agents. Monolayer (2D) cell culture approaches are limited by the lack of a physiologic context, while three-dimensional human tissue systems, although taking into consideration cell–cell and cell–extracellular matrix interactions, are still not demonstrative of the OA heterogeneity [16,17]. Preclinical animal models represent a more complex system, but are not totally representative of the human physiopathology, frequently leading to the failure of therapeutic responses in a later stage of the drug validation process.

In the search of effective drugs that might prevent or slow down the development of the disease, natural products derived from plants and marine organisms, remain a source of new molecular entities for the treatment of chronic inflammatory related diseases, including osteoarthritis [18,19]. Dietary supplements, of natural and synthetic origin, representing a nutritional and health benefit, were already associated with OA in human clinical trials. Although most were associated with OA pain relief, some were shown to modify the inflammatory OA process, by balancing anabolic and catabolic joint events, and promoting the synthesis of structural articulation precursors [20,21,22].

Among natural products, those containing phenolic rings, such as the flavonoids and some meroterpenoids, are usually provided of interesting biological activities, and have been shown to modulate cytokines such as tumor necrosis factor-α (TNFα), interleukin-1β (IL-1β) and interleukin-6 (IL-6), with a crucial role in chronic inflammatory and autoimmune diseases [23]. Some terpenoids based drugs are already available in the pharmaceutical market such as artemisinin and paclitaxel (Taxol^®^), acting as antimalarial and anticancer drugs, respectively [24].

In recent years, a series of meroterpenoids isolated from the brown alga *Cystoseira usneoides* have been shown to exhibit anti-inflammatory and antioxidant activities, by reducing the secretion of pro-inflammatory cytokines and downregulating the expressions of COX-2 and iNOS enzymes in THP-1 activated macrophages [25,26,27,28]. Among those, amentadione (YP) (Figure S1) showed radical-scavenging activity and demonstrated a significant role in reducing the production of TNFα in LPS-stimulated THP-1 human macrophages [26]. These results led us to further investigate the anti-inflammatory action of this pure marine compound and its potential as a novel cartilage protective agent in an OA context. For this purpose, we designed an OA preclinical pipeline consisting of an in vitro 2D-cell based system followed by an ex vivo explant-based and co-culture OA models. Our aim is to evaluate the potential protective effect of YP in the interplay between mineralization and inflammatory processes involved in OA development and progression.

## 2. Results

### 2.1. YP Acts as an Anti-Inflammatory Agent in the Articular OA Cell System Model

To evaluate the anti-inflammatory potential of YP in the mineralization and inflammatory processes involved in OA development and progression, a first screening was performed on THP-1 macrophages (THP-1 MOM). The inflammatory response of lipopolysaccharide (LPS)-induced THP-1 MOM was significantly reduced by pre-treatment with YP in a dose dependent manner (Figure S2a), as previously reported [25,26]. Additionally, the increased levels of TNFα production in calcium/phosphate (Ca/P) hydroxyapatite (HAP)-treated THP-1 MOM, confirmed the induction of a pro-inflammatory response [29], which was reduced by YP pre-treatment (Figure S2b). Cell proliferation assays were performed to confirm that tested HAP and YP did not affect THP-1 MOM cell viability (Figure S3). Based on these results, YP was further tested on a previously established articular OA cell system, consisting of human chondrocytes and synoviocytes primary cell cultures [30].

**Figure 1:**
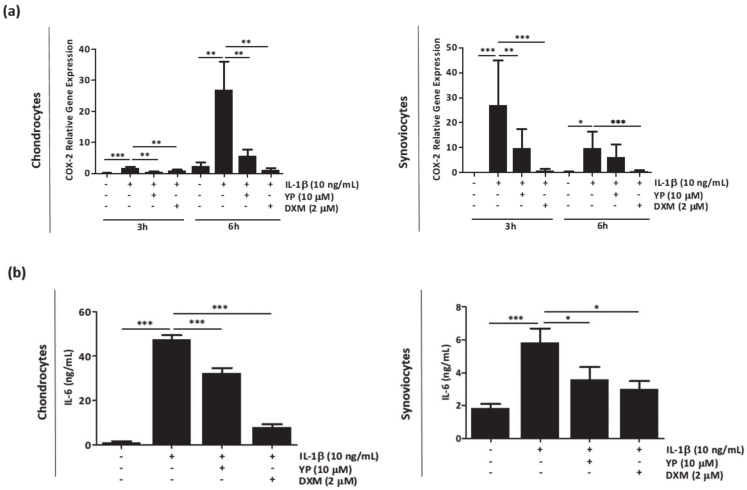
Amentadione (YP) reduces the levels of inflammatory markers in articular-derived cells stimulated with IL-1β (**a**,**b**). Primary chondrocytes and synoviocytes were pre-treated with 10 μM YP for 24 h, followed by stimulation with 10 ng/mL IL-1β during different time points. (**a**) Relative gene expression of the inflammatory marker COX-2 was determined by qPCR, at 3 h and 6h post IL-1β stimulation in chondrocytes and synoviocytes. (**b**) Levels of IL-6 in cell culture media 6 h post IL-1β stimulation, determined by ELISA. Cells treated with 2 μM dexamethasone (DXM) were used as a positive anti-inflammatory control. Data are presented as means of at least three independent experiments. All graphs show mean ± SD. One-way ANOVA and multiple comparisons were achieved with the Dunnett’s test. Statistical significance was defined as *p* ≤ 0.05 (*), *p* ≤ 0.005 (**) and *p* ≤ 0.0005 (***).

**Figure 2:**
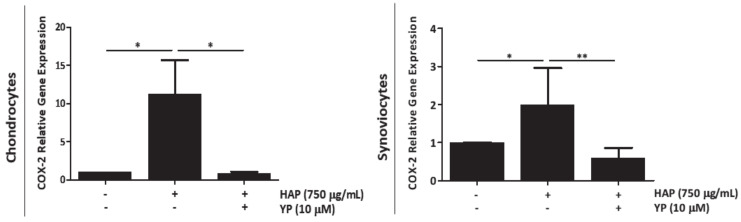
YP downregulates the inflammatory marker COX-2 in articular-derived cells stimulated with hydroxyapatite (HAP). Primary chondrocytes and synoviocytes were pre-treated with 10 μM YP for 24 h, followed by stimulation with 750 µg/mL HAP during 6 h. Relative gene expression of COX-2 was determined by qPCR, at 6 h post HAP stimulation in chondrocytes and synoviocytes. Data are presented as means of two independent experiments, with duplicates. All graphs show mean ± SD. One-way ANOVA and multiple comparisons were achieved with the Dunnett’s test. Statistical significance was defined as *p* ≤ 0.05 (*) and *p* ≤ 0.005 (**).

Human synoviocytes and chondrocytes primary cells were pre-treated with YP for 24 h followed by IL-1β (Figure 1) and HAP (Figure 2) stimulation. The effect of YP was determined by measuring gene expression of the inflammatory marker cyclo-oxygenase-2 (COX-2) and levels of IL-6 released into the cell culture media. Pre-treatment with YP followed by IL-1β stimulation resulted in a significant downregulation of COX-2 and decreased levels of IL-6 in both type of cells, relative to non-treated cells (Figure 1a,b). No cytotoxicity was observed in chondrocytes and synoviocytes, when treated with different YP concentrations (Figure S4).

Interestingly, a similar upregulation of the inflammatory marker COX-2 was observed in chondrocytes and synoviocytes treated with HAP, which was reduced by YP pre-treatment (Figure 2). No cytotoxicity was observed in chondrocytes and synoviocytes, when treated with different HAP concentrations (Figure S5).

These results demonstrate a promising anti-inflammatory effect of YP in the articular OA cell system model, through downregulation of inflammatory genes either when stimulated with IL-1β or treated with the mineralizing agent HAP.

### 2.2. YP Modulates Cartilage Homeostasis under Mineralizing Conditions in an Ex-Vivo Cartilage Explant Model

**Figure 3:**
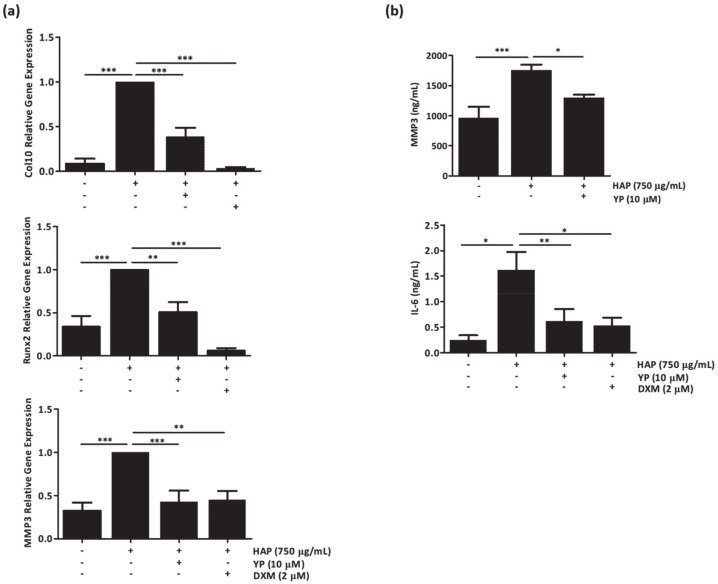
YP downregulates cell differentiation, extracellular matrix degradation and pro-inflammatory markers associated with osteoarthritis (OA) in the ex vivo cartilage explant model under HAP stimulation. Human cartilage explants were pre-treated with 10 μM YP for 24 h, followed by 72 h of 750 μg/mL HAP stimulation. Relative gene expression of Col10, Runx2 and MMP3 was determined by qPCR (**a**), and levels of MMP3 and IL-6 accumulation in the culture media were determined by ELISA (**b**). DXM indicates treatments with 2 μM dexamethasone. Data are presented as means of at least three independent experiments. All graphs show mean ±SD. One-way ANOVA and multiple comparisons were achieved with the Dunnett’s test. Statistical significance was defined as *p* ≤ 0.05 (*), *p* ≤ 0.005 (**) and *p* ≤ 0.0005 (***).

Since cartilage is the main affected tissue in OA, and ectopic mineralization is a known trigger of several joint alterations, including inflammation and cell differentiation, ultimately leading to cartilage degradation, cartilage tissue explants were first selected as an experimental model to evaluate ex vivo the effect of YP in response to HAP stimulation. Cartilage explants used in all experimental conditions were classified as normal- to early-OA tissues through the modified Mankin score [31]. Histological analysis revealed a smooth surface, a normal and uniform structural organization, and a normal to slight reduction in matrix staining, with Mankin total score ranging from 1 to 4 in the 1/13 modified Mankin scale (Table S1). The results showed that HAP treatment significantly upregulated collagen-10 (Col10), runt-related transcription factor-2 (Runx2) and matrix metalloproteinase-3 (MMP3) relative to control explants (Figure 3a), with simultaneous increased accumulation of MMP3 and the inflammatory marker IL-6 (Figure 3b). Pre-treatment of human cartilage explants with YP and further HAP stimulation, resulted not only in a significant down-regulation of the referred differentiation and ECM-related genes (Figure 3a), but also in decreased levels of the inflammatory marker IL-6 and the catabolic OA marker MMP3, responsible for ECM degradation (Figure 3b).

### 2.3. YP Function as a Protective Agent against Cartilage Deterioration under OA Promoting Conditions in an Explant-Based Co-Culture OA Model

**Figure 4:**
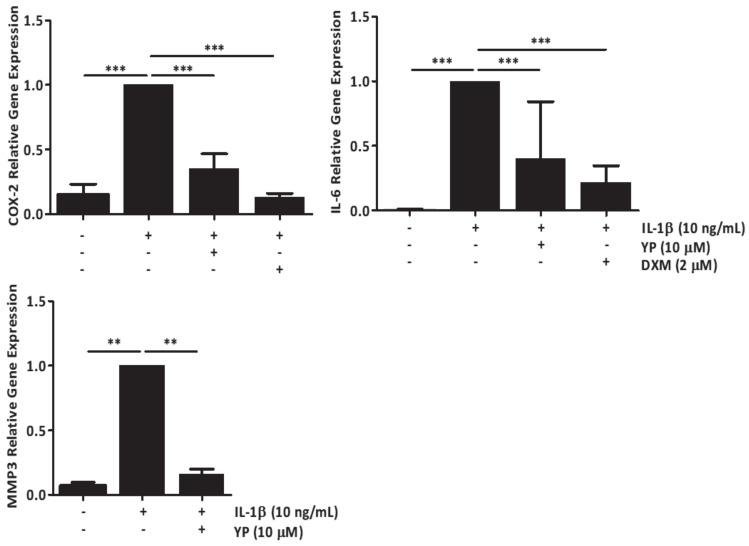
YP downregulates pro-inflammatory and ECM degradation markers associated with OA in the explant-based co-culture OA model under inflammatory stimulation with IL-1β. Cartilage explants co-cultured with human primary synoviocytes were pre-treated with 10 μM YP for 24 h, followed by 24 h of 10 ng/mL IL-1β stimulation. Relative gene expression of COX-2, IL-6 and MMP3 in cartilage explants were determined by qPCR. DXM indicates treatments with 2 μM dexamethasone. Data are presented as means of at least three independent experiments. One-way ANOVA and multiple comparisons were achieved with the Dunnett’s test. All graphs show mean ±SD. Statistical significance was defined as *p* ≤ 0.005 (**) and *p* ≤ 0.0005 (***).

**Figure 5:**
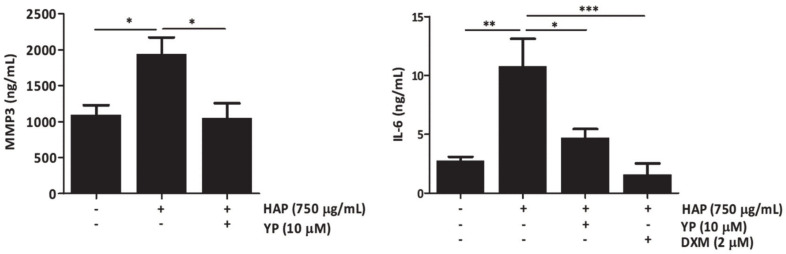
YP decreases the production of ECM degradation and pro-inflammatory markers in the explant-based co-culture OA model under mineralizing conditions. Cartilage explants co-cultured with human primary synoviocytes were pre-treated with 10 μM YP 24 h, followed by 72 h of 750 μg/mL HAP stimulation. Levels of MMP3 and IL-6 accumulation in the co-culture media were determined by ELISA. DXM indicates treatments with 2 μM dexamethasone. Data are presented as means of at least three independent experiments. One-way ANOVA and multiple comparisons were achieved with the Dunnett’s test. All graphs show mean ±SD. Statistical significance was defined as *p* ≤ 0.05 (*), *p* ≤ 0.005 (**) and *p* ≤ 0.0005 (***).

Since in the joint environment cartilage and synovial membrane are known to be involved in an interrelated and complex crosstalk affecting cartilage integrity and driving OA progression, an ex vivo explant-based co-culture OA model was developed and used to study the effects of YP in cartilage. Human cartilage explants were co-cultured with primary human synoviocytes and treated with IL-1β (Figure 4) and HAP (Figure 5) to simulate inflammatory and mineralizing conditions. Increased gene expression of COX-2, IL-6 and MMP3 in the co-culture cartilage explants treated with IL-1β, clearly indicated an induction of inflammatory reactions and ECM degradation at cartilage tissue level, which were consistently diminished in cartilage pre-treated with YP (Figure 4).

Additionally, increased levels of MMP3 and IL-6 in the cell culture media of co-culture cartilage explants treated with HAP demonstrated the interplay between mineralization and inflammation with consequent increased levels of inflammatory and ECM degrading markers, which were decreased with the YP pre-treatment (Figure 5).

Overall, considering the effects of YP at cartilage tissue level, evaluated using the cartilage explants and the explant-based co-culture models, the results suggest that YP exerts a cartilage protective effect, by reducing inflammatory reactions and preventing chondrocyte differentiation towards extracellular matrix mineralization and degradation.

### 2.4. YP Downregulates NF-kB Expression and Inhibits Ikbα Phosphorylation in Primary Chondrocyte Cells

Since YP was able to downregulate several pro-inflammatory mediators known to be directly regulated by the nuclear factor-κB (NF-kB) signaling pathway, we investigated whether the anti-inflammatory action of YP was due to its effect on NF-kB transcription and phosphorylation of its inhibitor IkBα.

**Figure 6:**
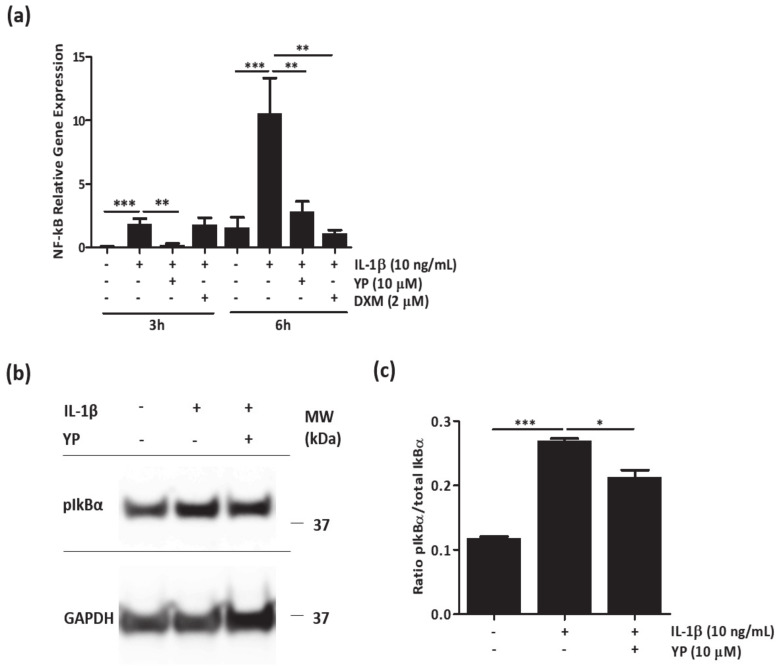
YP downregulates NF-kB expression and inhibits IkBα phosphorylation in IL-1β-stimulated primary articular chondrocytes. (**a**) Relative gene expression of NF-kB was determined by qPCR at 3 h and 6 h post 10 ng/mL IL-1β stimulation. Data is presented as mean of three independent experiments. (**b**) Total protein extracts of chondrocytes cultured in untreated conditions, stimulated with 10 ng/mL IL-1β for 30 min, and pre-treated with YP (μM) followed by 30 min of 10 ng/mL IL-1β treatment, were analyzed by Western blot to detect pIkBα. Position of relevant molecular mass marker (kDa) is indicated on the right side and GAPDH was used as loading control. (**c**) The pIkBα ratio (pIkBα/total IkBα) was determined in total protein extracts of chondrocytes cultured in control conditions (Ctr); 30 min of IL-1β (10 ng/mL) treatment; and pre-treated with YP for 24 h followed by 30 min of IL-1β stimulation (YP), by measuring the content of total and pIkBα with the specific InstantOne ELISA assay kit. Data is presented as mean of two out of four representative experiments. All graphs show mean ±SD. One-way ANOVA and multiple comparisons were achieved with the Dunnett’s test. Statistical significance was defined as *p* ≤ 0.05 (*), *p* ≤ 0.005 (**) and *p* ≤ 0.0005 (***).

In human primary articular chondrocytes, pre-treated with YP for 24 h followed by IL-1β stimulation, NF-kB expression was significantly downregulated at all-time points tested (Figure 6a). To determine the effect of YP in IkBα phosphorylation (pIkBα), known to precede NF-kB nuclear translocation, an initial experiment was performed to determine the optimal time point of pIkBα under IL-1β stimulation. Western blot analysis of chondrocyte protein extracts indicated increased levels of pIkBα from 30 min to 60 min of IL-1β treatment (Figure S6). Based on that, detection of pIkBα in chondrocytes pre-treated with YP for 24 h followed by 30 min IL-1β stimulation suggests a reduction of pIkBα in the YP treated chondrocytes relatively to the untreated and IL-1β stimulated cells (Figure 6b). Specific ELISA assays measuring pIkBα and total IkBα at 30 min shown that YP treatment reduces the ratio of pIkBα/total IkBα (Figure 6c), strongly indicating an effect of YP on IkBα phosphorylation.

These results demonstrate an anti-inflammatory effect of YP in articular chondrocytes, by downregulation of NF-kB expression, and inhibition of its activation through modulation of IkBα phosphorylation, and consequent downregulation of several NF-kB-related target genes.

## 3. Discussion

In this study we demonstrated that YP, a meroterpenoid isolated from the brown alga *C. usneoides* [26], is able to decrease inflammation, cell differentiation and extracellular matrix (ECM) degradation in different osteoarthritis in vitro/ex vivo OA model systems. By using a pipeline with increasing complexity, from 2D monolayer cultures of THP-1 macrophages, primary chondrocytes and synoviocytes, to ex vivo culture of human cartilage explants and a newly developed OA explant-based co-culture model, YP consistently promoted a protective effect under pro-inflammatory and mineralizing stimuli. These results bring new evidences on the health benefits of YP as a protective OA agent by attenuating cartilage degrading processes under known OA promoting stimuli, with consequent cartilage maintenance promoting effects, with potential therapeutic application.

In OA, cartilage loss and synovial membrane inflammation are two major factors responsible for disease progression and associated outcomes. Complex and interconnected molecular events of cartilage homeostasis disruption associated to inflammation known to fuel cartilage degradation, are recognized as crucial for disease development and important targets for therapeutic approaches [32]. Cartilage degradation is associated with chondrocytes differentiation leading to apoptosis and deposition of a mineralized extracellular matrix, which in turn contributes to loss of ECM integrity and inflammation [33]. In fact, although the pathways involved in the crosstalk between inflammation and cartilage degradation are still not completely clarified, mineralizing and inflammatory events occur in a close related manner during OA progression [30]. BCP and calcium pyrophosphate (CPP) crystals, consistently associate with the early stage of OA and have a pathogenic role in the development and rapid progression to end-stage OA [11,12]. BCPs have been found in the synovial fluid and membrane, and cartilage from OA patients [34], and associated with the activation of macrophages, synovial fibroblasts and articular cells, resulting in increased cell proliferation and production of pro-inflammatory cytokines and MMPs [35,36]. In concordance, our results show an inflammatory response to hydroxyapatite stimulation in all tested OA models, similar to those obtained with the classical inflammatory cytokine IL-1β, and to previously reported effects in OA cell models [30,37]. Of particular relevance, at cartilage level, treatment with hydroxyapatite induced overexpression of Col10 and Runx2, indicative of triggered chondrocyte differentiation towards hypertrophy and calcification. In addition, up-regulation of COX-2 and IL-6, widely known to be associated with joint inflammation, and MMP3, a major responsible for ECM degradation, clearly demonstrate the detrimental potential of calcification in OA. This is in line with recent data showing that BCP upregulate IL-6 in in vivo murine OA models, which in turn induced the expression of genes involved in calcification, promoting BCP formation and potentiating a vicious cycle [38]. Increased levels of BCP and IL-6 were also associated with cartilage degradation through the induction of matrix-degrading enzymes activity in chondrocytes [38]. In another study, calcium-phosphate complexes were shown to induce MMP3 and MMP13, which in turn, promoted the release of calcium and phosphate through degradation of the ECM calcified cartilage, in a positive loop [39]. Additionally, the effect of IL-1β on cartilage is known to reflect not only the catabolic effect of aggrecanases and MMPs upregulation, but also the downregulation of chondrogenic extracellular matrix synthesis [40,41]. In concordance, our results showed that IL-1β induced an overexpression of MMP3. Overall, our results clearly demonstrated the potentialities of the developed ex vivo explant-based co-culture OA model to study the interplay between cartilage degradation and inflammation, reflecting early molecular events leading to subsequent phenotypic cartilage alterations, of critical value in drug development for potential anti-osteoarthritic compounds such as YP.

YP has previously shown to have anti-inflammatory properties associated with the inhibition of TNFα in LPS-activated human macrophages [26]. In the present study, we demonstrated that YP was able to counteract inflammation, cell differentiation and ECM degradation, induced not only by IL-1β but also by hydroxyapatite, in all OA models, including primary articular cells, cartilage explants and ex vivo explant-based co-culture systems. These effects were demonstrated at multiple levels. Through downregulation of master players involved in pro-inflammatory reactions, such as NF-kB, COX-2 and IL-6, and the ECM catabolic marker MMP3, YP is directly contributing to preserve cartilage homeostasis, by avoiding ECM disruption and cartilage collapse. Similarly, the capacity to downregulate crucial genes involved in chondrocyte differentiation such as Col10 and Runx2, suggests YP as an inhibitor of chondrocytes hypertrophic differentiation. The resulting decrease of apoptosis and ECM mineralization, indirectly contributes to a consequent decrease of pro-inflammatory reactions, ultimately preserving cartilage homeostasis. Although our studies were not directed to evaluate the effect of YP as a structural cartilage-modifying drug, its capacity to inhibit early molecular events leading to joint deterioration, suggests YP as a potential disease modifying OA drug, worth to be further investigated.

Additionally, this YP protective role might represent a promising alternative to the anti-inflammatory drugs commercially available to manage symptomatology associated with OA and chronic autoimmune and inflammatory diseases, mostly based on NSAIDs target to inactivate COX enzymes (COX-1 and COX-2) [42], or biologics targeting crucial pro-inflammatory cytokines such as TNFα and IL-1β [43,44]. Although some effectiveness has been shown in slowing inflammatory reactions, the growing list of adverse side effects and the high percentage of patients presenting no response to these treatments, clearly demonstrate the urgent need for safer and more effective anti-inflammatory drugs. In this field, natural derived products, such as YP, have been considered as promising and valid alternatives. Some examples are the tetracyclic triterpenoid glycoside Ginsenoside Rb1 (G-Rb1) and curcumin, which have shown both in vitro and in vivo the capacity of targeting the production of several pro-inflammatory species and promoting the synthesis of anti-inflammatory mediators, with cartilage protective effects [45,46,47,48].

Considering the pivotal role of NF-kB as a major regulator of inflammation, many strategies have been developed to block NF-kB signaling in a variety of inflammatory disease settings [49]. Although in the context of OA these strategies are still in their infancy, the crucial role of NF-kB signaling mediating inflammatory responses, but also the hypertrophic conversion of articular cartilage chondrocytes, leading to ECM damage and cartilage destruction, is of paramount importance in the disease context [50,51]. Our results demonstrate that YP is able to downregulate NF-kB expression and decrease IkBα phosphorylation in chondrocytes, strongly suggesting that YP cartilage protective properties are associated, at least in part, with the inhibition of NF-kB nuclear translocation and consequent decreased activation of catabolic pathways, including expression of cytokines and chemokines, inflammatory mediators, matrix degrading enzymes, and regulators of chondrocytes differentiation. In agreement, YP treatments consistently decreased levels of COX-2 and IL-6, MMP3, Col10 and Runx2 in cartilage tissue, clearly demonstrating the potential of YP in ameliorating cartilage homeostasis and integrity, a good rationale for the exploitation of YP in the treatment of OA.

## 4. Materials and Methods

### 4.1. Isolation of Amentadione (YP)

The meroditerpenoid amentadione (YP) was isolated from the brown alga *Cystoseira usneoides* collected off the coast of Tarifa (Spain) as previously described [26]. Briefly, the frozen alga was extracted with methanol and after evaporation of the solution under reduced pressure, the aqueous residue was extracted with diethyl ether. The resulting extract was subjected to column chromatography (CC) on silica gel (70–230 mesh) (Merck KGaA, Darmstadt, Germany) eluting with a mixture of *n*-hexane/diethyl ether (50:50, *v*/*v*), then diethyl ether, mixtures of chloroform/methanol (90:10 and 80:20, *v*/*v*), and finally methanol. The fraction that eluted with chloroform/methanol (90:10, *v*/*v*) was further separated by CC on silica gel using as eluents mixtures of *n*-hexane/ethyl acetate (50:50 to 30:70, *v*/*v*), then ethyl acetate, and finally methanol. The compound YP was obtained by reversed phase HPLC separation of selected subfractions using as eluent methanol/water (70:30, *v*/*v*). HPLC separations were performed on a LaChrom-Hitachi apparatus (Merck), equipped with Kromasil 100-5C18 columns (250 × 10 mm, 5 μm or 250 × 4.6 mm, 5 μm) (Hichrom, Reading, UK), using an RI-71 differential refractometer or L-7400 UV detector (Merck). The pure compound YP was identified as amentadione [52] by NMR and HRMS analysis (Figure S1) and the negative optical rotation. NMR spectra were recorded on an Agilent 500 spectrometer (Agilent Technologies, Santa Clara, CA, USA), HRMS spectra were obtained on a Waters SYNAPT G2 spectrometer (Waters, Milford, MA, USA), and optical rotation was measured on a JASCO P-2000 polarimeter (JASCO, Tokyo, Japan).

### 4.2. Cell Culture

Primary human chondrocytes and synoviocytes were commercially acquired (chondrocytes, Lonza, Visp, Switzerland; synoviocytes, ECACC, Sigma-Aldrich, St. Louis, MO, USA) and obtained from human tissue explants using well-defined methodology [53,54]. Both cell types were cultured in Advanced Dulbecco’s Modified Eagle’s Medium (Adv DMEM) (Invitrogen, Carlsbad, CA, USA) supplemented with 10% (*v*/*v*) of heat-inactivated Fetal Bovine Serum (FBS, Sigma-Aldrich), 1 mM of l-glutamine (l-Gln, Invitrogen) and 1% (*v*/*v*) of penicillin-streptomycin (PS, Invitrogen). THP-1 cell line was kindly given by Dr. Santos (CBME, University of Algarve, Faro) and was cultured according to ATCC instructions in RPMI Growth Medium (RPMI 1640 with l-glutamine (Lonza)) containing 10% heat-inactivated FBS (invitrogen) and 1% PS. Differentiation into THP-1 macrophage (THP-1 MOM) cells was achieved by culturing THP-1 cells in 25 ng/mL phorbol 12-myristate 13-acetate (PMA) (Sigma) in complete RPMI for 48 h. All cell cultures were maintained at 37 °C in a humidified atmosphere containing 5% CO_2_, and experiments were performed on confluent cells.

### 4.3. Inflammatory Assays in Monolayer Cells

THP-1 MOM (1 × 10^6^ cells/mL) were cultured in 500 µL of complete RPMI supplemented with different amentadione (YP) concentrations (2.5, 5 and 10 µM) in dose-dependence experiments and with 10 µM YP in subsequent experiments, or with 2 µM dexamethasone (DXM), during 24 h. After, 100 ng/mL of lipopolysaccharides (LPS) or synthetic hydroxyapatite nano-crystals (HAP) (Sigma) (750 μg/mL) were added to the culture media for another 24 h or 72 h, respectively. Confluent chondrocytes and synoviocytes were cultured in 1 mL of Adv DMEM supplemented with 10 µM of YP or 2 µM of DXM during 24 h, and further treated with: 10 ng/mL of interleukin-1β (IL-1β) for 3 and 6 h, or for 30 min in the assay for pIKBα content analysis; 750 μg/mL HAP for 6h. Control cells were cultured with respective media without any treatment. At determined time points, cell culture media were collected for ELISA analysis and cells harvested for RNA extraction.

### 4.4. Cell Proliferation

THP-1 MOM cells seeded in 96-well plates at 2 × 10^5^ cells/well, and confluent chondrocytes and synoviocytes, were cultured in their respective growth media and supplemented with different concentrations of YP and HAP. Cell viability was determined at appropriate time points using the CellTiter 96 cell proliferation assay (Promega, Madison, WI, USA), following manufacturer’s instructions.

### 4.5. Cartilage Collection and Tissue Explants Preparation

Knee articular cartilage was obtained from osteoarticular cuts performed on the femoral and tibial sides, from eight patients (4 male and 4 female, aged 71.5 ± 5.9 years) who had undergone arthroplasty surgeries at Hospital Particular do Algarve (Faro, Portugal). This study was approved by the ethics committee of the hospital, and written informed consent was obtained from all the participants. All principles of the Declaration of Helsinki of 1975, as revised in 2000, were followed. Macroscopically normal full-depth cartilage slices were removed in sterile conditions using a scalpel, collected in complete Adv DMEM media, and incubated for 24 h, at 37 °C, in a humidified atmosphere containing 5% CO_2_, for tissue equilibration before preparation of tissue explants. After equilibration, 2 mm diameter and 1.71 ± 0.70 mm thickness cartilage explants, were obtained using a 2 mm biopsy punch (Integra-Miltex). Samples of the initial cartilage explant tissues were fixed in 4% PFA for histological evaluation.

### 4.6. Cartilage Explants Assays

Cartilage explants (8–10 per well), were plated in a 12 well plate and cultured at 37 °C, in a humidified atmosphere containing 5% CO_2_, in 1 mL of complete Adv DMEM media supplemented with 10 µM of YP or 2 µM DXM for 24 h, and then treated with HAP (750 μg/mL), for further 72 h. As controls, explants were cultured without treatment. At the end of each experiment, cartilage explants were collected, washed twice with PBS, immediately processed for RNA extraction as described below, and the cell culture media collected for ELISA assays.

### 4.7. Co-Culture Assays

Cartilage explants (10 per well), were plated in a 12 well plate and co-cultured with synoviocytes in a transwell system (6.5 mm insert diameter, 3.0 µm polyester membrane, Corning Incorporated Life Sciences), in 1.8 mL of complete Adv DMEM, at 37 °C, in a humidified atmosphere containing 5% CO_2_.

To evaluate the effect of YP, co-cultures were supplemented with 10 µM of YP or 2 µM DXM for 24 h, followed by treatment with IL-1β (10 ng/mL) during 24 h, or HAP (750 μg/mL) during 72 h. Cartilage explants were collected as described above for RNA extraction, and cell media collected for ELISA analysis.

### 4.8. RNA Extraction, cDNA Amplification and Quantitative Real-Time PCR (qPCR)

Cartilage tissue was immediately snap-frozen and manually grounded to powder in liquid nitrogen. Cells and tissue lysis was performed in a proportion of 1 mL of 4 M guanidine thiocyanate solution per 10^7^ cells or 100 mg cartilage tissue, thoroughly mixed and passed 10 times through a 22G needle. Total RNA was further extracted as described by Chomczynski and Sacchi [55]. Briefly, homogenates were sequentially mixed with 2 M sodium citrate pH 4, phenol pH 4.2 and chloroform/isoamyl alcohol. After centrifugation, total RNA present in the aqueous phase was precipitated with isopropanol, redissolved in 4 M guanidine thiocyanate solution, reprecipitated in isopropanol, washed with 75% ethanol and resuspended in Sigma water. RNA concentration was determined by spectrophotometry at 260 nm (Nanodrop 1000, Thermo Scientific, Waltham, MA, USA). RNA was then treated with RQ1 RNase-free DNase (Promega, Madison, WI, USA) and reverse-transcribed using the qScipt cDNA SuperMix (Quanta bio, Beverly, MA, USA) according to manufacturer’s recommendations. Quantitative real-time PCR reactions were performed using the CFX connect, Real time System (Bio-Rad, Richmond, CA, USA), SoFast Eva Green Supermix (Bio-Rad, Richmond, CA, USA), 300 nM of forward and reverse gene-specific primers for genes of interest (Table S2), and a 1:5 cDNA dilution. The following PCR conditions were used: initial denaturation/enzyme activation step at 95 °C for 13 min, 50 cycles of amplification (one cycle is 15 s at 95 °C and 30 s at 68 °C). Fluorescence was measured at the end of each extension cycle in the FAM-490 channel. Levels of gene expression were calculated using the comparative ∆∆Ct method, and normalized using gene expression levels of glyceraldehyde-3-phosphate dehydrogenase (GAPDH), with the iQ5 software (BioRad).

### 4.9. ELISA Assays

The cell culture media were used for the quantification of TNFα (Peprotech), IL-6 (Peprotech) and MMP3 (Life Technologies) following the manufacture’s protocols.

### 4.10. Histological Evaluation

Paraffin-embedded cartilage tissue sections were processed at the Histopathology Department of Centro Hospitalar e Universitário do Algarve (CHUA, Faro) and used for histological assessment. Cartilage grading of initial tissue samples was conducted based on modified criteria originally established by Mankin et al., and the specimens were analyzed for abnormalities in structure, cellularity and matrix staining, based on hematoxylin-eosin (HE, Bio-Optica, Milano, Italy), safranin-O (SO)/Fast Green (Sigma-Aldrich, Steinheim, Germany) and toluidine blue (Merck, Darmstadt, Germany) stainings [56,57]. Four tissue sections from each sample were analyzed.

### 4.11. Protein Extraction and Quantification

Total protein from chondrocyte inflammatory assays and YP treatments was obtained by extraction with RIPA buffer (50 mM Tris HCl pH 8, 150 mM NaCl, 1% NP-40, 0.5% sodium deoxycholate, 0.1% SDS) for 1 h, at 4 °C, with agitation, followed by a centrifugation at 16× *g* for 15 min at 4 °C. Protein concentration was assessed using Micro BCA kit (Thermo Scientific), according to the manufacturer’s instructions.

### 4.12. Electrophoresis and Western Blot

Aliquots of 20 µg of total protein extracts were size separated in a 4–12% (*w*/*v*) gradient polyacrylamide precast gel containing 0.1% (*w*/*v*) SDS (NuPage, Invitrogen, Carlsbad, CA, USA) and transferred onto a nitrocellulose membrane (Biorad, Richmond, CA, USA). Detection of pIKBα and GAPDH was performed through overnight (O/N) incubation with the pIKBα pSer32 ABfinity Rabbit Monoclonal antibody (2.5 μg/µL, Thermo Fisher, Waltham, MA, USA) and anti-GAPDH polyclonal antibody (1:500, Santa Cruz Biotechnology). Detection was achieved using Goat anti-rabbit IgG horseradish peroxidase-conjugated secondary antibody and Western Lightning Plus-ECL (PerkinElmer Inc., Waltham, MA, USA). Image acquisition was obtained using an IQ LAS 4000 mini biomolecular imager.

### 4.13. Determination of Total and Phosphorylated IkBα

Total IkBα and phosphorylated IkBα (pIkBα) were determined in chondrocyte cell lysates, using the InstantOne ELISA assay kit (Invitrogen) according to the manufacturer’s protocol.

### 4.14. Statistical Analysis

Each independent experiment (*n*) was performed with different primary cell culture batches and cartilage from distinct patients. Replicates within an individual experiment were performed using the same batch of cells and cartilage from a single patient. Data are presented as mean ± standard deviation (SD). Multiple *t* tests were used for comparison between two groups. For more than two groups significance was determined using one-way analysis of variance (ANOVA) with comparison between groups by Dunnett test. Statistical significance was defined as *p* ≤ 0.05 (*), *p* ≤ 0.005 (**) and *p* ≤ 0.0005 (***).

## Supplementary Materials

The following are available online at https://www.mdpi.com/1660-3397/18/12/624/s1, Figure S1: Chemical structure, NMR and HRMS data of amentadione (YP); Figure S2: YP reduces the inflammatory response of THP-1 macrophages (THP-1 MOM) stimulated with LPS and hydroxyapatite (HAP); Figure S3: Viability of THP-1 macrophage cells (THP-1 MOM) exposed to different concentrations of amentadione (YP) for 24 h and exposed to different concentrations of HAP for 72 h; Figure S4: Viability of primary human chondrocytes and synoviocytes exposed to different amentadione (YP) concentrations for 24 h; Figure S5: Viability of primary human chondrocytes and synoviocytes exposed to different hydroxyapatite (HAP) concentrations for 72 h; Figure S6: Indication on the time point with increased pIkBα after IL-1β stimulation. Table S1: Modified Mankin score used for histological evaluation of human cartilage explants; Table S2: Gene-specific primers used for gene expression analysis by qPCR.
