# Supplementary material for: Amentadione from the Alga Cystoseira usneoides as a Novel Osteoarthritis Protective Agent in an Ex Vivo Co-Culture OA Model
Source: Mar Drugs. 2020 Dec 7;18(12):624. doi: 10.3390/md18120624 (PMC7762386; doi:10.3390/md18120624)
Supplement: Supplementary file 1 [file marinedrugs-18-00624-s001.pdf]

Amentadione from the Alga *Cystoseira usneoides* as a Novel Osteoarthritis Protective Agent in an *Ex Vivo* Co-Culture OA Model

Nuna Araújo [1], Carla S. B. Viegas [1,2], Eva Zubía [3], Joana Magalhães [4,5,6], Acácio Ramos [7], Maria M. Carvalho [7], Henrique Cruz [7], João Paulo Sousa [7], Francisco J. Blanco [4,5], Cees Vermeer [8] and Dina C. Simes [1,2,*]

1 Centre of Marine Sciences (CCMAR), University of Algarve, Faro, 8005-139, Portugal; naraujo@ualg.pt (N.A.); caviegas@ualg.pt (C.S.B.V.)
2 GenoGla Diagnostics, Centre of Marine Sciences (CCMAR), University of Algarve, Faro, 8005-139, Portugal; caviegas@ualg.pt (C.S.B.V.); dsimes@ualg.pt (D.C.S.)
3 Department of Organic Chemistry, Faculty of Marine and Environmental Sciences, University of Cadiz, Puerto Real (Cádiz), 11510, Spain; eva.zubia@uca.es
4 Unidad de Medicina Regenerativa. Grupo de Investigación en Reumatología (GIR). Instituto de Investigación Biomédica de A Coruña (INIBIC). Complejo Hospitalario Universitario de A Coruña (CHUAC). Sergas, A Coruña, Spain; joana.cristina.silva.magalhaes@sergas.es (J.M.); fblagar@sergas.es (F.J.B.)
5 Universidade da Coruña (UDC), Agrupación Estratégica CICA-INIBIC. A Coruña, Spain
6 Centro de Investigación Biomédica en Red (CIBER), Madrid, Spain
7 Department of Orthopedics and Traumatology, Hospital Particular do Algarve (HPA), Gambelas-Faro, 8005-226, Portugal; acacioramos@gmail.com (A.R.); mariamiguelfc@gmail.com (M.M.C.); cruzhenrique@hotmail.com (H.C.); jprsous@gmail.com (J.P.S.)
8 Cardiovascular Research Institute CARIM, Maastricht University, 6229 EV Maastricht, The Netherlands; cees.vermeer@outlook.com
* Correspondence: dsimes@ualg.pt

Received: date; Accepted: date; Published: date

Figure S1: Chemical structure, NMR and HRMS data of amentadione (YP).

Figure S2: YP reduces the inflammatory response of THP-1 macrophages (THP-1 MOM) stimulated with LPS and hydroxyapatite (HAP).

Figure S3: Viability of THP-1 macrophage cells (THP-1 MOM) exposed to different concentrations of amentadione (YP) for 24 h and exposed to different concentrations of HAP for 72 h.

Figure S4: Viability of primary human chondrocytes and synoviocytes exposed to different amentadione (YP) concentrations for 24 h.

Figure S5: Viability of primary human chondrocytes and synoviocytes exposed to different hydroxyapatite (HAP) concentrations for 72 h.

Figure S6: Indication on the time point with increased pIkBα after IL-1β stimulation.

Table S1: Modified Mankin score used for histological evaluation of human cartilage explants.

Table S2: Gene-specific primers used for gene expression analysis by qPCR.

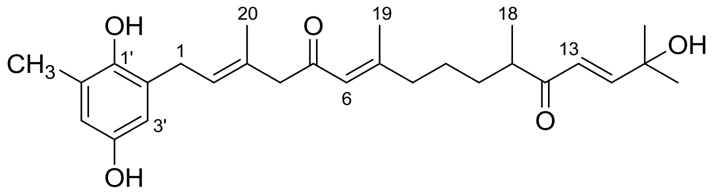

Amentadione (YP): yellowish oil; ^{1}H NMR (CD_3OD, 500 MHz) δ 6.94 (1H, d, *J* = 15.8 Hz, H-14), 6.39 (2H, br s, H-3′ and H-5′), 6.35 (1H, d, *J* = 15.8 Hz, H-13), 6.20 (1H, br s, H-6), 5.46 (1H, br t, *J* = 7.4 Hz, H-2), 3.31 (2H, d, overlapped with the solvent signal, H-1), 3.10 (2H, s, H-4), 2.85 (1H, m, H-11), 2.15 (3H, s, 6′-Me), 2.13 (2H, t, *J* = 7.2 Hz, H-8), 2.07 (3H, d, *J* = 1.2 Hz, Me-19), 1.69 (3H, br s, Me-20), 1.64 (1H, m, H-10a), 1.41 (2H, m, H-9), 1.32 (1H, m, H-10b), 1.32 (6H, s, Me-16 and Me-17), 1.06 (3H, d, *J* = 6.9 Hz, Me-18); ^{13}C NMR (CD_3OD, 125 MHz) δ 206.7 (C, C-12), 202.2 (C, C-5), 160.8 (C, C-7), 155.2 (CH, C-14), 151.5 (C, C-4′), 146.5 (C, C-1′), 131.3 (C, C-3), 130.8 (C, C-2′), 129.3 (CH, C-2), 127.7 (C, C-6′), 125.6 (CH, C-13), 123.6 (CH, C-6), 115.9 (CH, C-5′), 114.6 (CH, C-3′), 71.3 (C, C-15), 56.2 (CH_2, C-4), 45.0 (CH, C-11), 42.0 (CH_2, C-8), 33.7 (CH_2, C-10), 29.9 (CH_2, C-1), 29.30 (CH_3, Me-16), 29.29 (CH_3, Me-17), 26.1 (CH_2, C-9), 19.4 (CH_3, Me-19), 17.1 (CH_3, Me-18), 16.9 (CH_3, 6′-Me), 16.6 (CH_3, Me-20); HRESIMS *m*/*z* 441.2643 $[M\text{-}H]^-$ (calcd. for $C_{27}H_{37}O_5$ 441.2641).

Figure S1. Chemical structure, NMR and HRMS data of amentadione (YP**)**.

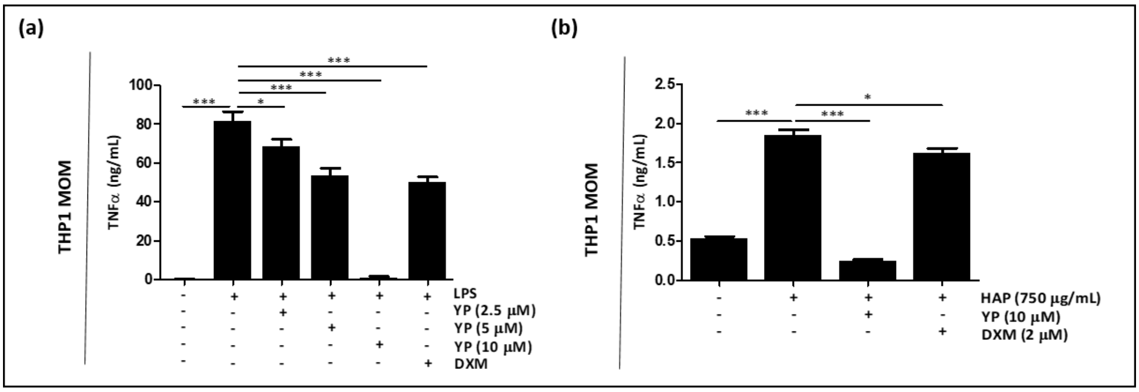

Figure S2. YP reduces the inflammatory response of THP-1 macrophages (THP-1 MOM) stimulated with LPS (a) and hydroxyapatite (HAP) (b). Levels of TNFα in cell culture media of THP-1 MOM pre-treated with different concentrations of YP for 24 h, followed by exposure to 100 ng/ml LPS for additional 24h (a) and to 750 µg/mL HAP for 72 h (b), determined by ELISA. Control (Ctr) corresponds to culture media of non-treated cells, and cells treated with 2 µM dexamethasone (DXM) were used as a positive anti-inflammatory control. Data are presented as means of at least three independent experiments. All graphs show mean ±SD. One-way Anova and multiple comparisons were achieved with the Dunnett's test. Statistical significance was defined as *p*≤0.05 (*), *p*≤0.005 (**) and *p*≤0.0005 (***).

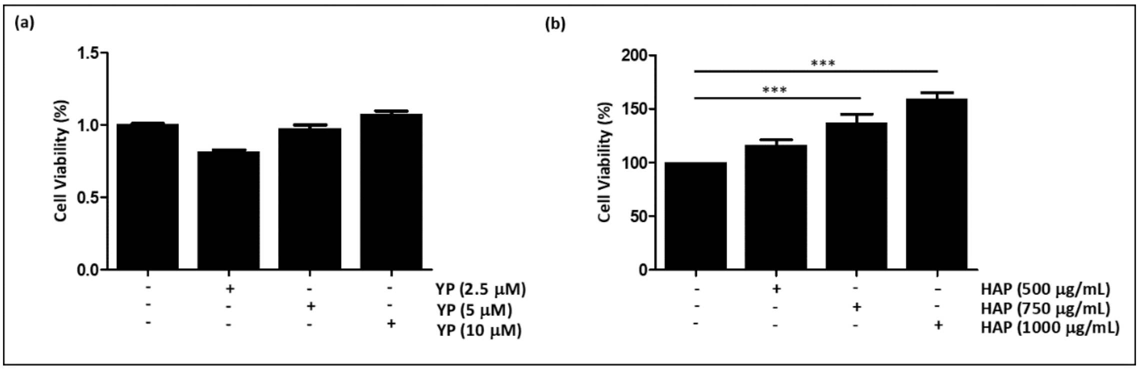

Figure S3. Viability of THP-1 macrophage cells (THP-1 MOM) exposed to different concentrations of amentadione (YP) for 24 h (a) and HAP for 72 h (b). Control (Ctr) corresponds to cell culture media of non-treated cells. Graph shows mean ±SD. One-way Anova and multiple comparisons were achieved with the Dunnett's test. Statistical significance was defined as p≤0.0005 (***).

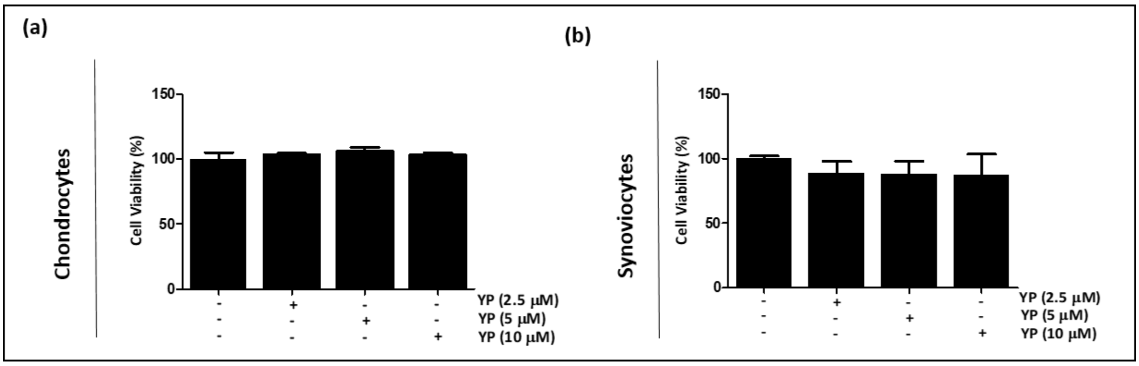

Figure S4. Viability of human primary chondrocytes (a) and synoviocytes (b) exposed to different amentadione (YP) concentrations for 24 h. Control (Ctr) corresponds to culture media of non-treated cells.

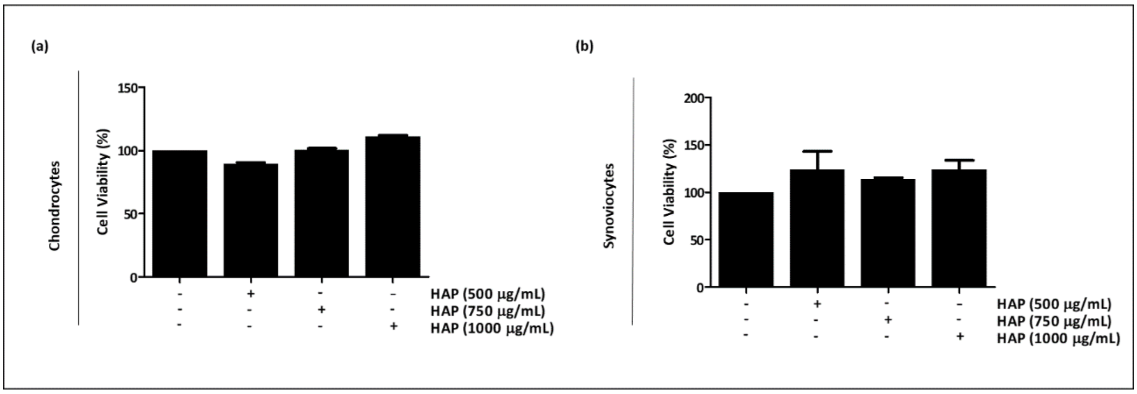

Figure S5. Viability of human primary chondrocytes (a) and synoviocytes (b) exposed to different hydroxyapatite (HAP) concentrations for 72 h. Control (Ctr) corresponds to culture media of non-treated cells.

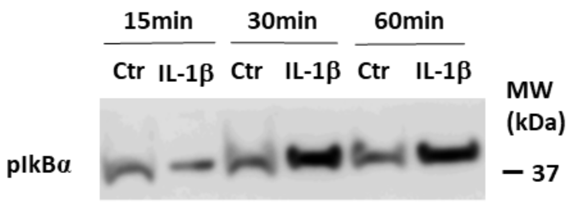

Figure S6. Indication on the time point with increased pIkBα after IL-1β stimulation. 20 g of total protein extracts of chondrocytes cultured in control (Ctr) and treated with 10 ng/mL IL-1β for different time points, were analysed by Western blot to detect pIkBα. Position of relevant molecular mass marker (kDa) is indicated on the right side.

Table S1. Modified Mankin score used for histological evaluation of human cartilage explants, using hematoxylin-eosin (HE), Safradin-O (SO) and Fast Green as staining

ID Sample	Mankin Score			Mankin Total Score
	Structure	Cellularity	Matrix Staining	
1	0	0	1	1
2	1	0	2	3
3	1	1	2	4
4	0	0	4	4
5	0	0	1	1
6	0	0	4	4
7	2	0	1	3
8	2	0	2	4

Table S2. Gene-specific primers used for gene expression analysis by qPCR.

Gene	Primer Designation	Sequence (5´ to 3’)
GAPDH	GAPDH_F	AAGGTGAAGGTCGGAGTCAACGGA
GAPDH_R		TCGCTCCTGGAAGATGGTGATGGG
COX2	COX-2_F	TGGTCTGGTGCCTGGTCTGATGATGT
COX-2_R		GCCTGCTTGTCTGGAACAACTGCTCA
NF-kB	NF-kB_F	GCAATCATCCACCTTCATTCTCAACTT
NF-kB_R		CCTCCACCACATCTTCCTGCTTAG
Col10	Col10_F	AGCTGCCAAGGCACCATCTCCA
Col10_R		AGTGGGCCTTTTATGCCTGTGGGC
MMP3	MMP3_F	CGTGGCAGTTTGCTCAGCCTATCC
MMP3_R		GCACTTCGGGATGCCAGGAAAGGT
Runx2	Runx2_F	TCCGCAGGTCACTACCAGCCACC
Runx2_R		GGTGTCACTGTGCTGAAGAGGCTGT
IL6	IL6_F	AAGCAGCAAAGAGGCACTGGCAGAA
IL6_R		CTGCACAGCTCTGGCTTGTTCCTCAC
